# Sarcopenia Is a Cause and Consequence of Metabolic Dysregulation in Aging Humans: Effects of Gut Dysbiosis, Glucose Dysregulation, Diet and Lifestyle

**DOI:** 10.3390/cells11030338

**Published:** 2022-01-20

**Authors:** James W. Daily, Sunmin Park

**Affiliations:** 1 Department of R & D, Daily Manufacturing Inc., Rockwell, 28138 NC, USA; jdaily3@yahoo.com; 2 Department of Food & Nutrition, Obesity/Diabetes Center, Hoseo University, Asan 31499, Korea

**Keywords:** skeletal muscle mass, inflammation, glucose metabolism, gut microbiome, short-chain fatty acids, gut microbiota-muscle axis

## Abstract

Skeletal muscle mass plays a critical role in a healthy lifespan by helping to regulate glucose homeostasis. As seen in sarcopenia, decreased skeletal muscle mass impairs glucose homeostasis, but it may also be caused by glucose dysregulation. Gut microbiota modulates lipopolysaccharide (LPS) production, short-chain fatty acids (SCFA), and various metabolites that affect the host metabolism, including skeletal muscle tissues, and may have a role in the sarcopenia etiology. Here, we aimed to review the relationship between skeletal muscle mass, glucose homeostasis, and gut microbiota, and the effect of consuming probiotics and prebiotics on the development and pathological consequences of sarcopenia in the aging human population. This review includes discussions about the effects of glucose metabolism and gut microbiota on skeletal muscle mass and sarcopenia and the interaction of dietary intake, physical activity, and gut microbiome to influence sarcopenia through modulating the gut–muscle axis. Emerging evidence suggests that the microbiome can regulate both skeletal muscle mass and function, in part through modulating the metabolisms of short-chain fatty acids and branch-chain amino acids that might act directly on muscle in humans or indirectly through the brain and liver. Dietary factors such as fats, proteins, and indigestible carbohydrates and lifestyle interventions such as exercise, smoking, and alcohol intake can both help and hinder the putative gut–muscle axis. The evidence presented in this review suggests that loss of muscle mass and function are not an inevitable consequence of the aging process, and that dietary and lifestyle interventions may prevent or delay sarcopenia.

## 1. Introduction

Sarcopenia is a progressive loss of muscle mass and function associated with aging or immobility [1]. More specifically, sarcopenia is defined as low muscle strength, reduced quantity and quality of muscle mass, and decreased physical performance [2]. Stated another way, “Sarcopenia is a progressive, generalized skeletal muscle disorder involving the combination of loss of muscle mass and loss of muscle function and/or muscle strength, as well as loss of muscle performance” [3]. Age-related muscle wasting diseases can be categorized as either sarcopenia or cachexia. Cachexia is muscle wasting that is associated with disease processes such as cancer. Sarcopenia is muscle loss due to the aging process and is perhaps classified as lean or obese sarcopenia depending on the fat mass of the sarcopenic individual [4]. Sarcopenia appears to be inextricably intertwined with glucose metabolism, and the maintenance of muscle mass in addition to the balance of insulin sensitivity and insulin secretion plays a critical role in maintaining glucose homeostasis [1]. However, insulin resistance is modulated by complex interactions with various factors, including body composition, gut microbiota, and various nutrient intakes. Increasing insulin resistance due to aging, obesity, inflammation, and oxidative stress elevates insulin secretion, eventually leading to chronic hyperglycemia and decreased lifespan [5].

**Figure 1:**
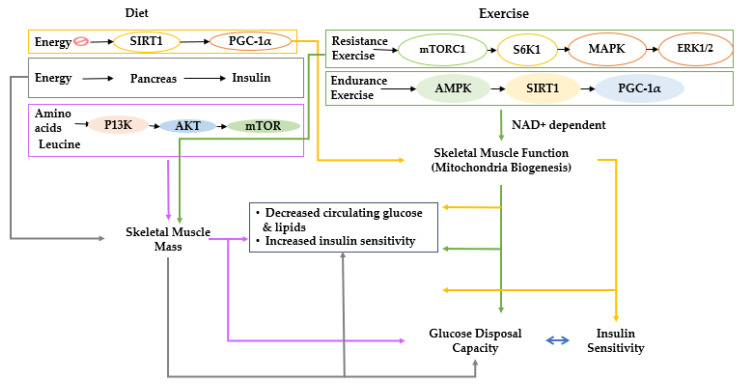
Proposed interactions of diet and lifestyle with skeletal muscle and glucose metabolism. Effects of diet and lifestyle on skeletal muscle mass and function and the impact on blood glucose regulation are explained; diet and lifestyle are shown to exert effects on muscle mass and function, which have interactive effects on glucose metabolism. Sirt 1, NAD-dependent deacetylase sirtuin-1; PGC-1α, peroxisome proliferator-activated receptor-gamma coactivator 1α; mTORC1, mechanistic target of rapamycin C1; S6K1, ribosomal protein S6 kinase beta-1; MAPK, mitogen-activated protein kinase; ERK1/2, extracellular signal-regulated kinases; AMPK, AMP-activated protein kinase; PI3K, phosphoinositide 3-kinase; AKT; NAD^+^, nicotinamide adenine dinucleotide.

Many scientists have investigated which tissues are primarily responsible for insulin resistance. Skeletal muscle has been identified as one of the responsible target tissues. Skeletal muscle tissues are insulin-dependent, and they are the most significant contributor to insulin-dependent glucose disposal [5]. Consequently, decreased skeletal muscle mass and impaired muscle glucose metabolism are possible contributors to type 2 diabetes and vice versa [5,6]. Skeletal muscle is believed to begin during the third decade of life and gradually decreases the capacity for muscle glucose utilization, increasing susceptibility to type 2 diabetes [7]. Type 2 diabetes increases sarcopenia risk and has a bidirectional relationship [5]. The balance between anabolism and catabolism regulates skeletal muscle mass. Anabolism is stimulated by growth factors, including androgens, insulin-like growth factor-I (IGF-I), insulin, and some myokines such as irisin, myonectin, decorin, and fibroblast growth factor (FGF)-21 [8]. Catabolism-related signaling pathways counterbalance anabolism in response to stimuli such as glucocorticoids and proinflammatory cytokines (Figure 1) [9]. These endogenous factors influence the Akt/mammalian target of rapamycin (mTOR), SMAD, autophagy, and nuclear factor kappa-light-chain-enhancer of activated B cells (NF-κB) signaling pathways to modulate mitochondriogenesis, myogenesis, and muscle atrophy [10]. Among those pathways, IGF-1/insulin signaling is directly involved in insulin sensitivity, and it indirectly affects the NF-κB signaling pathways to modulate skeletal muscle mass [11]. Therefore, the IGF-1/insulin and NF-κB signaling pathways are closely associated with maintaining skeletal muscle mass.

Many human studies have explored possible interventions to prevent the loss of skeletal muscle mass and insulin sensitivity during aging [12,13]. Sedentary lifestyles are a primary factor responsible for decreases in skeletal muscle mass and insulin sensitivity, and physical exercise is strongly linked to lower sarcopenia risk, especially in older adults [14]. The causal relationship between other lifestyle-related factors and skeletal muscle mass remains controversial because most research has been conducted using observational studies [14,15]. However, smoking and excessive alcohol drinking may have inverse associations with skeletal muscle mass [14,15]. Adequate energy and protein intake helps protect against declines in muscle mass and other specific nutrient intakes, including ω-3 fatty acid, nicotinamide adenine dinucleotide (NAD^+^) precursors, vitamin D, anti-inflammatory and antioxidant containing foods, and dietary fiber, are also involved in maintaining muscle mass by modifying insulin sensitivity, oxidative stress, and inflammation [14,15]. Although the exact mechanisms have not been identified, they modulate biogenesis and degradation of muscle tissues [16].

Recent studies have shown that the gut microbiota can impact various organs to influence the initiation and progression of sarcopenia [17,18,19]. The gut microbiota compositions may impact glucose and fat metabolism in skeletal muscle tissues [17]. Gut microbiota modulates lipopolysaccharide (LPS) production, short-chain fatty acids (SCFA), and various metabolites that affect host metabolism, including skeletal muscle tissues and their function through the gut–muscle axis [18,19]. Although there is no direct evidence of the association between sarcopenia and distinct gut microbiota composition in the elderly, the potential relationship between skeletal muscle mass and gut microbiota has been reported [20]. Increasing evidence has demonstrated that the intake of probiotics and prebiotics modulates skeletal muscle mass and function through modulating gut permeability and the gut–muscle axis [21]. This review examines how skeletal muscle, glucose regulation, and the gut microbiota may interact with each other and with diet and lifestyle to promote healthy aging and prevent sarcopenia.

## 2. Overall View of Muscle Metabolism and the Factors That Control Muscle Growth

As the population ages, there is an increasing burden of frailty characterized by bone loss, often resulting in osteoarthritis and fractures and muscle loss, sometimes progressing to a clinical diagnosis of sarcopenia [22]. The mechanical loads imposed on bone due to muscle contraction are critical for stimulating bone growth and maybe a propounding factor in developing degenerative bone diseases of aging as people become more sedentary [22]. The decreased physical activity is also believed to be an essential factor in the lifestyle changes that lead to sarcopenia [23]. Furthermore, it has been recently observed that low numbers of circulating osteoprogenitor cells, an indicator of mesenchymal stem cells in bone marrow, are associated with frailty and sarcopenia [24]. Therefore, it is likely that both muscle loss and bone loss exacerbate each other and are crucial factors in developing frailty during aging.

Skeletal muscle plays a critical role not only in glucose and lipid metabolism but also in endocrine and paracrine activities [25]. Genetic and environmental factors and disease status are involved in maintaining skeletal muscle mass, which interacts with various organs, including bone, adipose tissue, liver, heart, and brain [25,26]. Skeletal muscle mass is a net result of both catabolic and anabolic metabolisms of myocytes. Muscle loss is related to reduced satellite cell recruitment and anabolic hormonal signaling, protein oxidation, inflammation, and developmental factors [27]. Muscle breakdown is involved in elevated oxidative stress, degenerative neuromuscular junction, and hyperglycemia, in addition to muscle mass loss [27,28]. However, the molecular mechanisms have not been clearly characterized [26].

Skeletal muscles and bone metabolisms are closely related during development and growth, and their loss is a major clinical problem for the elderly population and a cause of falls and fragility fractures [29]. Their interactions are related to the endocrinological and metabolic interconnection of muscle and bone and adiposity in the bone marrow and muscles [30]. Muscle atrophy, osteoporosis, and fatty infiltration into bone marrow and skeletal muscle should be managed to prevent sarcopenia-induced falls and bone fractures [30]. Skeletal muscles comprise a metabolic network linked to other organs by myokines produced and released by muscle fibers [29]. Myokines are classified as myostatin, myostatin-binding proteins, including follistatin and decorin, interleukins (IL) including IL-6, IL-7, and IL-15, insulin-like growth factor 1, and other myokines, including irisin and osteoglycin. Myostatin acts as a negative regulator of myogenesis; follistatin promotes muscle growth by inhibiting the binding of myostatin to its receptor, irisin, and IL-6 promote oxidative metabolism in myofibers. IGF-1 promotes muscle growth, and osteoglycin enhances cell–cell contact during myogenesis. They are involved in not only muscle differentiation and growth but also bone resorption and formation. Follistatin, IGF-1, decorin, irisin, and osteoglycin are involved in bone formation, whereas IL-15 regulates bone resorption [31]. Thus, muscle–bone communication plays an essential role in sarcopenia.

Sarcopenia sometimes accompanies body fat increase and vice versa. Muscle loss and fat accumulation can create a vicious cycle to increase metabolic dysfunction via complicated interaction of proinflammatory cytokines, oxidative stress, mitochondrial dysfunction, insulin resistance, and mitochondrial dysfunction [32]. Increased insulin resistance elevates ectopic fat accumulation, and it promotes the production of proinflammatory cytokines that inhibit muscle production and increase muscle catabolism [33]. Increased adipocyte mass and dysfunction result in elevated fatty acid flux from adipose tissues, contributing to fat accumulation in the liver and skeletal muscles. The fat accumulation elevates adipokine secretion and low-grade inflammation in the body, which develops insulin signaling impairment and mitochondrial dysfunction in the skeletal muscles, resulting in muscle atrophy [34]. Therefore, fat and glucose metabolism are closely associated with maintaining skeletal muscle mass.

## 3. Age-Related Interactive Effects of Impaired Glucose Metabolism and Sarcopenia

### 3.1. Skeletal Muscle Mass Loss during Aging

Skeletal muscle mass has been reported to be positively associated with a metabolically healthy phenotype in a large population study [35]. The benefits were valid for both skeletal muscle mass and muscle quality and were associated with having less than two components of metabolic syndrome [35,36]. Unfortunately, one of the most consistent physiological features of the aging process is a gradual but progressive loss of skeletal muscle mass and function [23]. Age-related loss of skeletal muscle mass begins at around age 30 and is a consistent feature of the aging process [37]. However, the rapidity and severity of the progression of muscle are highly variable and may be modulated by dietary and lifestyle factors. It is often difficult to precisely define the degree of muscle loss and compare the differences among people due to how muscle loss presents among individuals and differences in assessment methods [38]. However, the most severe muscle loss is termed sarcopenia and has become officially recognized as a disease state in recent years, and was assigned an International Diagnostic Classification Code (ICD-10-CM code M62.84) [39].

The progression of muscle loss is usually accompanied by various metabolic changes that manifest during different stages of the aging process and may be involved in changes in body composition. Sarcopenia is strongly linked to metabolic diseases and frailty [40]. Although muscle loss is associated with metabolic diseases such as type 2 diabetes, the mechanisms involved are not fully understood. The lack of clarity is partly due to the complex interactions between muscle and glucose regulation. However, it is known that insulin released to lower blood glucose concentrations also stimulates muscle growth. Muscle, in turn, is the primary site of insulin-stimulated glucose uptake. Decreased skeletal muscle quantity and quality can be devastating for glucose management since muscle is responsible for more than 80% of insulin-stimulated glucose disposal [41]. In addition to age-related loss of muscle mass, the function of the remaining muscle also declines with age, resulting in losses of muscular strength and endurance that surpasses that which can be attributed to loss of muscle mass alone [42].

### 3.2. Sarcopenia and Glucose Metabolism

The factors responsible for age-related declines in muscle mass and function are not fully understood, but anabolic resistance seems to be one of the significant contributors to sarcopenia [43]. However, the nature of anabolic resistance is somewhat obscure, and there are profound differences of opinion about its causes and effects. Many investigators believe that the anabolic signals from the two major anabolic stimuli, resistance exercise, and amino acid (especially leucine) consumption, are suppressed [44] due to a lessened activation of phosphoinositide 3-kinase (PI3K) activation, in turn, decreased AKT signaling as well as mTOR. PI3K/AKT/mTOR signaling is a primary stimulatory regulator of anabolic signals for muscle growth and inhibitor of muscle anabolism (Figure 1) [44]. The impairment of anabolic signaling pathways is a commonly held paradigm of anabolic resistance in skeletal muscle. However, other researchers have demonstrated that older people do not have a blunted anabolic response to amino acids and resistance exercise [45,46]. Instead, they have demonstrated that the elderly have an equal anabolic response as determined by muscle protein synthesis rate as well as an equal mammalian target of rapamycin complex 1 (mTORC1) response to stimuli such as leucine. This model suggests that a diminished availability of amino acids is responsible for anabolic resistance in the elderly, possibly due to poor absorption of amino acids from the diet. It would suggest that anabolic resistance can be overcome with higher protein intake, especially the amino acid leucine [47], and resistance exercise training. Both of the two explanations for anabolic resistance are supported by convincing evidence. Whichever explanation is correct, the fact remains that age is associated with a progressive loss of skeletal muscle mass and function.

In addition to the age-related loss of muscle mass, the functional impairment of existing muscle may also have critical consequences. The loss of muscle function is partly due to a shift in muscle fiber type to a greater percentage of type 1 (slow-twitch) fibers at the expense of type 2 (fast-twitch) fibers [48]. A study in men and women 21–87 years of age measured gene expression of muscle types in biopsy samples before and after exercise [49]. They found no age-related changes in myosin heavy chain (MHC) type 1 muscle proteins, but that respective expression levels of type IIa and type IIx declined by 14% and 10% per decade [33]. It was also observed that knee extensor strength declined with age, even when strength was normalized for muscle mass, demonstrating a decline in functionality of the remaining muscle [33]. A rather profound decrease in type 2 muscle fiber expression leads to a diminished reliance on glycolysis and glucose utilization in the cytosol of the cells and a high reliance on oxidative phosphorylation, contributing to increasing fat utilization in the mitochondria for energy metabolism [48]. This may be an unfortunate development, since simultaneous changes in skeletal muscle cells may make oxidative metabolism more difficult as the transitions to a greater reliance on oxidative metabolism occurs, as explained below. Furthermore, this diminished use of glucose for energy may exacerbate glucose dysregulation and accelerate the progression towards type 2 diabetes [50].

Oxidative metabolism occurs exclusively in the mitochondria of cells, and substantial research supports mitochondrial dysfunction as a central feature of aging skeletal muscle [51]. With aging, mitochondrial bioenergetics progressively decline, resulting in a simultaneous decline in the capacity to utilize oxygen. Analogous to the muscle itself, these changes in mitochondrial function appear to be due to both decreases in mitochondrial content and function [52]. Peroxisome proliferator-activated receptor γ coactivator (PGC)-1α is probably the most important, though not the exclusive, regulator of mitochondrial biogenesis, and its expression decreases in aged skeletal muscle at both the protein and mRNA level [53]. PGC1α is encoded by the peroxisome proliferator-activated receptor-gamma (PPARG; PPAR-γ) coactivator 1 alpha (PPARGC1A) gene. PGC-1α is downstream of several essential energy sensor genes that are important for the regulation of metabolic systems in humans, including AMP-activated protein kinase (AMPK), sirtuin 1 (SIRT1), and mitogen-associated protein kinase (p38 MAPK) [54]. The SIRT1 gene is often thought of as the longevity gene responsible for the anti-aging “French Paradox” effects of resveratrol from red wine [54]. SIRT1 is a deacetylase that is NAD^+^ dependent and appears to play a central role in regulating energy metabolism through its targets, which include PGC1-α and PPAR-γ [55]. Furthermore, NAD^+^ precursor supplementation has been demonstrated to increase mitochondrial biogenesis in the muscles of mice [56] and may work similarly in humans. Suppression of SIRT1 and, by extension, PGC-1α, can limit oxidative capacity and energy utilization, resulting in the elevation of circulating lipids, which can have a critical consequence on glucose metabolism [57].

Diabetes is associated with elevated blood glucose and elevated circulating lipids [57]. Furthermore, infusion of lipids into circulation can cause insulin resistance in both humans and experimental animals [58,59]. It notedly remains uncertain whether the loss of skeletal muscle mass and function are a cause or a consequence of diabetes. However, abundant evidence suggests both can cause and exacerbate the other. Furthermore, other factors may lead to impairments of both skeletal muscle and glucose regulation. Among the other factors, the influence of the gut microbiota may also greatly influence the regulation of glucose metabolism and skeletal muscle health, which could, in turn, affect glucose metabolism.

## 4. Glucose Metabolism and Gut Microbiota

**Figure 2:**
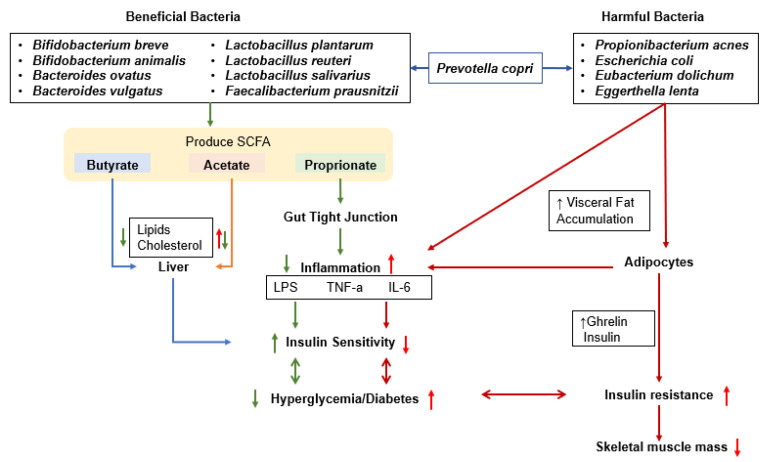
Proposed interaction of gut bacteria with glucose metabolism. Effects of gut microbiota and their products on the liver, gut cell wall, adipocytes, and inflammation as regulators of glucose metabolism are explained; gut bacteria can increase and decrease inflammation, either improving or exacerbating insulin resistance and muscle loss. LPS, lipopolysaccharide; TNF-α, tumor necrosis factor-α; IL-6, interleukin-6.

Genetic and environmental factors influence glucose metabolism, affecting both insulin secretion and insulin sensitivity [60,61]. Carbohydrate digestion and absorption, inflammation, nutrient intakes, and lifestyle factors such as alcohol consumption and exercise influence glucose homeostasis. The gut microbiome is an emerging factor that influences glucose metabolism by interacting with various factors and shows bidirectional symbiosis (Figure 2) [62]. Gut microbiota produces SCFA that may influence the host’s glucose metabolism by promoting the gut–liver, gut–skeletal muscle, gut–islet, and gut–brain axes [63]. These effects modulate insulin sensitivity, insulin secretion, and glucose disposal.

Bacteria use glucose as an energy source, and glucose supply is a significant factor for commensalism with the host and bacteria colonization. Gut bacteria can be categorized as having mutualism, commensalism, and parasitism, all of which can affect the host’s glucose metabolism [64]. Hyperglycemia of the host impairs tight junction expressions of the intestinal cells that increase pathogen-associated molecular patterns, including LPS and toll-like receptor ligands and bacteria translocation into the host cells [64]. As a result, diabetic patients are susceptible to infection and inflammation when exposed to pathogenic bacteria, and their infections cannot be easily cured since diabetic patients have defects in innate and adaptive immunity [65,66]. Furthermore, uncontrolled diabetes elevates the inflammatory response and oxidative stress, exacerbating infection [67].

## 5. Effects of the Microbiome on Muscle Mass and the Development of Sarcopenia

### 5.1. Physical Activity, Sarcopenia, and Gut–Muscle Axis

The gut microbiome is a menagerie of bacteria, archaea, fungi, viruses, and eukaryotic microbes that live as independent life forms in humans and other mammalian hosts [68]. The microbiota making up the microbiome can profoundly affect the host, potentially affecting almost all physiological systems [69]. It seems counter-intuitive that the microorganisms living in the intestinal tract can regulate such seemingly disconnected systems as skeletal muscle mass and function. However, in a 2017 review article, Ticinesi et al. [70], outlined the current animal and human research supporting a bidirectional gut–muscle axis associated with sarcopenia. First of all, it had been well established that after age 65, there is a decline in the abundance and diversity of the gut microbiota, especially taxa believed to contribute health benefits [71]. Several studies have confirmed a strong association between exercise and fitness and a healthy robust microbiome, suggesting that exercise may stimulate the growth of healthy gut microbes in addition to muscle increment [72,73]. Additionally, aerobic exercise has been shown to reduce age-induced reactive oxygen species (ROS) production to inhibit mitochondrial dysfunction and mitophagy, thereby preventing sarcopenia [74]. However, there has been limited direct evidence to show that gut microbiota can improve muscle mass or function [75]. Indirect evidence supporting gut microbiota benefits for skeletal muscle health includes a study that took stool samples from older adults (ages 70–85) with high physical function and samples from age-matched low physical function adults and fed the feces to germ-free mice [76]. The mice fed fecal materials from high-functioning elderly adults had significantly greater grip strength but not muscle mass and endurance. The increment of grip strength was associated with higher bacterial counts of *Prevotella* and *Barnesiella* bacteria in the intestines of mice fed the fecal samples [60]. Another mouse study saved mice feces before treating the mice with antibiotics to make them primarily germ-free and found that muscle fatigue increased and endurance decreased after the antibiotic treatment, but both were normalized in mice in which their own microbiomes were restored [77]. Therefore, mouse studies have shown that the absence of gut bacteria can cause impairment of skeletal muscle and that restoration of both mouse and healthy human bacteria can be used to restore muscle function in mice.

After the aforementioned review article, more studies have investigated the reciprocal effects of the microbiome on muscle mass. One such study compared elderly (ages 60–70) high-endurance athletes with same-aged subjects who met usual physical activity standards [78]. The most notable differences in gut bacteria between the groups were a much higher *Prevotella* and modestly lower *Bacteroides* counts in the high-endurance athletes, although the authors pointed out that the effect on *Bacteroides* is not consistent with some previous studies [78]. The differences in body composition included significantly lower BMI and total body fat and higher, but not significantly, muscle mass. The lack of a significant difference in muscle mass may not be surprising since they were high-endurance athletes, and endurance exercise is not known to stimulate muscle growth. However, it has been demonstrated that butyric acid-producing bacteria in the gut are beneficial for preserving muscle mass [79].

### 5.2. Gut Microbiota, Sarcopenia, and Gut–Muscle Axis

The changes in gut microbiota are involved with skeletal muscle mass by changing SCFA and inflammatory cytokines to modulate the gut–muscle axis. Qui et al. used an antibiotic cocktail to mostly eliminate the gut microbiota of 10-week-old C57BL/6 mice and determine the effects on body composition [80]. The antibiotic-treated mice experienced skeletal muscle atrophy linked to decreased ileal farnesoid X receptor (FXR)-FGF15 signaling and subsequently impaired skeletal muscle protein synthesis. Okamoto et al. investigated the effects of short-chain fatty acids produced by gut bacteria in mice [81]. They demonstrated that SCFA (acetate, butyrate, and propionate) were almost completely eliminated from fecal samples of antibiotic-treated mice, which had significantly decreased exercise endurance, but an infusion with acetate restored the exercise endurance in the mice. This study demonstrated that gut-produced short-chain fatty acids, primarily acetate, may be essential energy sources for muscle function. Nay et al. [17], also found that eliminating the gut bacteria using antibiotics impairs muscle function and endurance, partly due to muscle glycogen depletion [17]. It has also been shown that elite athletes increase both microbial diversity and abundance during extreme exercise [81] but mainly increase bacteria-produced SCFA [81,82,83,84]. This was demonstrated by a metagenomic (16S DNA) analysis of stool samples from elite athletes before and after running a marathon. After running, the most remarkable difference was a substantial increase in species of the *Veillonella* genus, which metabolize lactate into acetate and propionate [84]. A similar study in Olympic rowers found that the relative abundance of butyrate-producing bacteria increased, and insulin sensitivity was improved after prolonged rowing [82].

In 728 female twins, sarcopenia was inversely correlated with gut microbiota α-diversity and the relative abundance of *Faecalibacterium prausnitzii*, a well-known SCFA producer, whereas the relative abundances of *Eubacterium dolichum* and *Eggerthella lenta* had a positive correlation [85]. The evidence suggests that increasing the amount of SCFA producing bacteria may be an adaptation to increase SCFA for muscle fuel and perhaps decrease lactate accumulation [73]. Whereas the microbiome can improve muscle performance and endurance, the evidence that it can increase muscle mass is intriguing but not as strong. Furthermore, if the gut microbiome can regulate muscle mass, it is also unclear whether it is directly affected or mediated by interactions of the microbiome with other organs such as the gut–brain, gut–liver, gut–immune, and gut–organ axis. In a recent review, Zhao et al. suggested several ways that the gut microbiota could influence the development of sarcopenia [84], including impaired insulin signaling and mitochondrial disruption. However, inflammation was considered a significant cause of muscle degradation, known as “inflammaging”. Gut inflammation may result in the malabsorption of nutrients, such as amino acids, that are important for stimulating muscle biogenesis [84,86]. The concept of a gut–muscle axis is relatively new, and there is much to be learned. However, it does seem plausible that a healthy gut microbiome can be protective against age-related loss of muscle mass and function. Therefore, skeletal muscle mass may be related to gut microbiota composition, but gut microbiota associated with skeletal muscle mass remains unclear. However, convincing evidence does support that a gut microbiota–skeletal muscle axis functions by modulating SCFA, proinflammatory cytokines, and the autonomous nervous system involved in skeletal muscle mass regulation [75,87].

### 5.3. Myokines, Sarcopenia, and Gut–Liver–Muscle Axis

Myokines are released from myocytes in response to muscle contraction, and they are involved in muscle metabolism and other tissues, including adipocytes, liver, and brain, through their receptors [8]. Several known myokines, including myostatin, irisin, myonectin, FGF-21, decorin, IL-6, IL-15, and others, and their biological functions have not been well characterized. Some known characteristics involve myocyte proliferation, differentiation, growth, and atrophy. Irisin, myonectin, decorin, FGF-21, secreted protein acidic and cysteine-rich (SPARC), and brain-derived neurotrophic factor have a positive activity to increase muscle mass, but myostatin, IL-6, and IL-15 are involved in muscle atrophy [7]. Thus, the regulation of myokine activity can protect against sarcopenia. Gut microbiota is involved in regulating myokine function through changing SCFA, secondary bile acids, branched-chain amino acids (BCAA), endocannabinoids, and inflammatory cytokines [88].

Ponziani et al. [89], have investigated the relationship of the microbiome with sarcopenia in patients with cirrhosis of the liver. In that study, the sarcopenic patients were low in *Methanobrevibacter*, *Prevotella*, and *Akkermansia*, which are generally considered health-promoting, while they were rich in *Eggerthella*, which is considered to be indicative of frailty, and pathogenic bacteria such as *Klebsiella* [65]. The study concluded that “alterations in the gut–liver–muscle axis is associated with sarcopenia” in patients with cirrhosis of the liver. Furthermore, myokines are associated with liver function to influence muscle mass [90]. Among the liver cirrhosis patients, those having higher serum myostatin concentrations exhibit a poor survival rate, and high serum myostatin concentrations are associated with muscle loss with hyperammonemia to suppress protein synthesis [90]. Thus, myokines can act as one of the modulators of the gut–liver–muscle axis to change muscle mass.

Although *Prevotella* proportions are inversely associated with the feces of sarcopenic adults, *Prevotella* is also reported to be associated with increased inflammation and insulin resistance in previous studies [91,92], and its increase may not be directly related to skeletal muscle mass. *Prevotella* may grow faster in the gut under the host’s low energy intake and high energy expenditure conditions, regardless of dietary fiber intake. Overall, however, the evidence is suggestive but not conclusive that the gut microbiota can regulate skeletal muscle growth and function during normal human aging. Animal studies provide additional mechanistic evidence to support a role for the microbiome in optimizing muscle mass and function during aging.

## 6. BCAA Effects on Metabolism and Sarcopenia Host through the Gut–Muscle Axis

Supplementing older adults with loss of muscle mass and function with BCAA and vitamin D for 8 weeks has improved muscle mass and function [93]. A Japanese study has found that supplementing BCAA to liver cirrhosis patients prevents sarcopenia and fat accumulation in skeletal muscle [94]. The apparent inconsistency between BCAA, insulin resistance, and type 2 diabetes may be related to BCAA utilization due to the attenuation of insulin resistance in the body. High serum BCAA concentrations are linked to insulin resistance, obesity, and type 2 diabetes, although BCAA supplementation is beneficial for increasing energy expenditure and skeletal muscle synthesis by activating mTOC1 [95,96,97]. Brown adipose tissues utilize BCAA catabolism in the mitochondria for thermogenesis, increasing energy expenditure [96]. However, a defect in the BCAA clearance mediated by *SLC25A44* attenuates BCAA clearance to increase serum BACC concentration, suggesting inducing insulin resistance [96].

Gut microbiota is closely related to BCAA metabolism and is involved in their synthesis and degradation; however, their imbalance by gut microbiota raises serum BCAA concentrations, contributing to increased insulin resistance [95]. One study has demonstrated that insulin-resistant participants have elevated serum BCAA concentrations, which correlated to elevated gut microbiota involved in BCAA biosynthesis and a lower expression of BCAA transporters [98]. *Prevotella copri* is a primary species associated with elevated biosynthesis of BCAAs [95]. *Prevotella copri* activates epithelial cells to generate IL-6, IL-8, and CCL20 and stimulates toll-like receptor-2, leading to Th17 immune responses and neutrophil recruitment to induce mucosal and systemic inflammation such as seen in autoimmune diseases [99]. *Prevotella copri* is also associated with insulin resistance and inflammation to exacerbate glucose intolerance [99].

*Bacteroides vulgatus* is also reported to promote BCAA biosynthesis and increase insulin resistance [95], but reports about its involvement in insulin resistance and inflammation are inconsistent [100]. Japanese cardiovascular patients have a lower abundance of *B. vulgatus* and LPS contents in the feces [100]. Furthermore, *B. vulgatus* intake is associated with reducing atherosclerotic lesions in atherosclerosis-prone mice with decreasing LPS production by gut microbiota [100]. Consuming a BCAA depleted diet for 4 weeks reduced postprandial insulin secretion, promoted insulin signaling in white adipose tissues, and modulated fecal microbiota composition by reducing relative abundance of Firmicutes and increasing relative abundance of Bacteroides [101]. Therefore, gut microbiota generates and utilizes the BCAA, SCFA, inflammatory cytokines, and neurotransmitters to influence insulin sensitivity of the host by promoting gut–liver, gut–skeletal muscles, and gut–brain axis [87,102]. Different individuals have different gut microbiota, and they need to consume optimal diets to modulate the gut microbiota to promote insulin sensitivity.

## 7. The Modulation of Dietary Intake and Lifestyles and Gut Microbiome to Promote Skeletal Muscle Mass and Prevent Sarcopenia

### 7.1. Calorie and Fat Intake, Gut Microbiota, and Skeletal Muscle Mass

Research associating dietary intake with specific changes in the gut microbiota often produces inconsistent results. However, current research suggests that a dietary program that increases microbial diversity in the gut, particularly when rich in species that produce SCFA, is highly beneficial for healthy skeletal muscle aging [103]. Various dietary factors, including intakes of prebiotics, fermented foods, fat, protein, and carbohydrates, can modulate gut microbiota composition [104]. The changes in gut microbiota are associated with not only the availability of energy sources but also intestinal permeability, digestion capacity of carbohydrates and proteins, gastric acid, and bile acid secretion [105]. Muscle loss is linked to insulin resistance and inflammation associated with gut microbiota dysbiosis, contributing to increased intestinal barrier permeability, serum inflammatory cytokines, and insulin resistance [75].

Calorie restriction has been reported to lower the risk of metabolic diseases and increase health-span by initiating mitophagy in various tissues [106]. It can potentiate AMPK-SIRT1, cAMP response element-binding protein, brain-derived neurotrophic factor, and FGF2 and inhibit inflammation and ROS-related pathways [107]. However, it remains unclear for maintaining skeletal muscle mass. A calorie restricted diet for 8 weeks has been shown to modulate intestinal microbiota to increase *Lactobacillus* in rats [106]. Calorie restriction with high protein promotes the α-diversity of gut microbiota, but calorie restriction with high protein and typical protein diets enriches *Akkermansia* and *Bifidobacterium* and depletes *Prevotella* [108]. However, calorie restriction does not maintain or increase skeletal muscle mass, although it does maintain muscle strength [109]. Nevertheless, intermittent fasting has been reported to reduce fat mass and maintain or increase skeletal muscle mass by ameliorating oxidative stress and decreasing proinflammatory cytokines in human and animal studies [110,111,112,113]. Intermittent fasting reduces body fat mass, insulin resistance, and proinflammatory cytokines in adults; it also induces significant changes in gut microbiota communities, increases the production of short-chain fatty acids, and decreases the circulating levels of LPS in adults [110]. After intermittent fasting, the relative abundances of *Ruminococcus gnavus*, *Chitinophagaceae bacterium*, *Roseburia faecis*, *Paraburkholderia caribensis*, *Verrucomicrobiae bacterium Ellin516*, *Neisseria dentiae*, and *Streptococcus ferus* increase [110]. Intermittent fasting modulates gut microbiota to increase Lactobacillales and decrease Clostridales in Alzheimer’s disease-induced rats [111]. Therefore, intermittent fasting with a high protein diet may help maintain or increase skeletal muscle mass while modulating gut microbiota. Skeletal muscle mass can be maintained or increased by modulating gut microbiota with an optimal weight loss regimen.

### 7.2. Probiotic and Prebiotic Intakes, Gut Microbiota, and Skeletal Muscle Mass

Skeletal muscle mass and strength decrease from middle age to the elderly, and it is difficult to prevent the decrement of muscle mass and strength only with dietary intake. The effects of protein and amino acids, especially leucine, vitamin D, ω-3 fatty acids, antioxidants, magnesium, and probiotics, have been studied as nutritional interventions to prevent sarcopenia in the elderly [114]. However, their preventive activities are minimal, but supplementation with exercise may be beneficial [114]. In an animal study, long-term supplementation of *Lactobacillus plantarum* TWK10 (1.03 × 10^9^/kg/day) significantly increased the number of slow-twitch muscles in gastrocnemius muscle and grip strength [115]. In young adults, the 6-week TWK10 intake also improves endurance performance in a maximal treadmill running test [116]. Furthermore, heat-killed *Bifidobacterium breve* B-3 intake significantly promoted skeletal muscle mass and function with mitochondrial biogenesis by increasing phosphorylation of AMPK and activation of PGC-1α and cytochrome c oxidase in the rat soleus [117]. Administering the probiotic, *Lactobacillus reuteri* modulates the forkhead box N1 transcriptional factor, delays cachexia in a mouse model of cancer, and is positively associated with muscle mass [70,118]. Finally, a study supplementing with *Lactobacillus plantarum* showed that the probiotic improved exercise performance and endurance as well as body composition, with fat mass being decreased and muscle mass increased [119]. These results suggested that specific probiotic intakes may maintain and increase skeletal muscle mass, strength, and function in humans and experimental animals through altering the gut microbiota. However, these studies did not determine the changes in gut microbiota. After probiotics supplementation, the probiotic bacteria are often eliminated in the stool not colonized in the large intestines within 2 months, and they are colonized in the large intestines in less than 10% of persons [120]. However, probiotic intakes partially alter gut microbiota composition to influence host metabolism [120]. The changes in skeletal muscle mass with probiotic supplementation may be related to changes in gut microbiota.

Prebiotics play a critical role in the diversity and composition of gut microbiota. A few studies have been conducted to examine the effects of prebiotics on skeletal muscle mass and muscle strength through changing gut microbiota [121]. A study in elderly human subjects evaluated the effects of consuming a prebiotic supplement consisting of inulin and fructooligosaccharide, which would be expected to promote a healthy microbiome [122,123]. They reported that the subjects having the prebiotic had greater hand strength and endurance than control subjects, although they did not report the effects on the microbiome [122,123]. Vegan diets rich in dietary fibers are associated with rich *Prevotella* and high microbial diversity, and *Prevotella* has been associated with high-carbohydrate and high-fiber diets [70]. However, Korean adults with *Prevotella* enterotype have a rice-based diet with lower energy intake than other diet types [92]. *Prevotella* may grow faster in the gut under the condition of low energy intake and high energy expenditure for the host’s requirement regardless of dietary fiber intake. *Prevotella* is also associated with increased inflammation and insulin resistance in the previous studies [91,92]. Further studies need to determine the association between *Prevotella*, dietary fiber, and skeletal muscle mass.

Prebiotic intake modulates bacteria-producing SCFA to alter skeletal muscle mass and muscle function. SCFA promotes IGF-1 release from the host to act as an anabolic hormone to make skeletal muscle and reduce inflammation [70,73]. In experimental animals, prebiotics, mostly fructans, modulate the gut microbiota to influence body weight and fat mass by releasing gut hormone hormones [124]. SCFA, especially propionate and butyrate, stimulates intestinal gluconeogenesis to activate neural signaling that inhibits satiety and improves insulin sensitivity [124]. They act as ligands of G-protein coupled receptors and release satiety hormone production, including peptide YY (PYY) and glucagon-like peptide-1 and activate PPAR-γ dependent mechanisms. Propionate is a modulator of β-cell function. These activities of SCFA reduce body weight and adiposity, which may maintain or stimulate skeletal muscle mass. However, no study has shown that prebiotic intake increases skeletal muscle mass, but polyphenols and their secondary metabolites promote muscle mitochondrial function to activate protein synthesis in experimental animals and older adults.

## 8. Summary and Conclusions

Age-associated physiological changes commonly include losses of muscle mass and function, declines in microbiome quality and diversity, and disruption of glucose regulation. The simultaneous occurrence of these changes is probably not coincidental since interactions among them can cause many of the underlying defects associated with each, and dietary and lifestyle changes that occur with aging can also contribute to each condition. Muscle mass and function can be improved by physiological stimuli such as load-bearing exercise, protein consumption, especially the amino acid leucine, and improving anabolic hormone signaling, including insulin and SIRT-1. Insulin resistance and inflammation are linked to muscle mass loss, but it is not yet known if loss of muscle mass is a consequence or cause of insulin resistance and/or inflammation. However, increased circulating lipids due to decreased uptake in muscle could lead to insulin resistance, and insulin resistance in muscle could decrease anabolic signaling by insulin/IGF-1. Increased proinflammatory cytokines reduce PGC-1α activation, increasing oxidative stress and NF-κB activation to elevate further inflammation linked to muscle degradation. The gut microbiome is also associated with increasing and maintaining muscle mass through modulating the gut microbiome-muscle axis by producing SCFAs and proinflammatory cytokines. Gut dysbiosis induces a leaky intestinal wall that allows toxic bacterial metabolites to enter the host metabolism. SCFA, especially propionate and butyrate, may act as AMPK activator to stimulate PGC-1α and insulin/IGF-1 signaling to promote muscle biogenesis.

Human aging is associated with impaired glucose utilization, decreased muscle mass and function, and lessened gut microbial diversity. There is increasing evidence that muscle mass decrement is accelerated by its interactions with dietary and lifestyle changes that impact it. It has been demonstrated that muscle mass and function remain near youthful levels with both load-bearing and endurance exercise as well as adequate intakes of protein and specific amino acids. Optimal muscle function improves glucose disposal in the muscle, which assists glucose management, as do signals from the gut microbiome. Finally, non-digestible carbohydrate intake results in SCFA production that stimulates muscle growth and improves glucose regulation. Therefore, this review provides convincing evidence that a concerted program of specific dietary and lifestyle interventions can have synergistic effects on multiple mutually supportive physiological systems that may delay age-related physical deterioration and prevent sarcopenia in aging adults.
